# A protocol for a systematic review of research on managing behavioural and psychological symptoms in dementia for community-dwelling older people: evidence mapping and syntheses

**DOI:** 10.1186/2046-4053-2-70

**Published:** 2013-08-28

**Authors:** Daksha Trivedi, Claire Goodman, Angela Dickinson, Heather Gage, Jennifer McLaughlin, Jill Manthorpe, Kunle Ashaye, Steve Iliffe

**Affiliations:** 1 University of Hertfordshire, Hatfield, UK; 2 University of Surrey, Guildford, UK; 3 King’s College London, London, UK; 4 Hertfordshire Partnership University NHS Foundation Trust, Lister Hospital, Stevenage, UK; 5 University College London, London, UK

**Keywords:** Behavioural and psychological symptoms, Community dwelling, Dementia, Synthesis, Systematic review

## Abstract

**Background:**

Non-cognitive behavioural and psychological symptoms of dementia affect up to 90% of people with dementia during the disease course and result in distress, increased carer burden, high service utilization and unwanted moves to care homes. Research has focused on long-term settings and has not considered people with dementia living at home and at different stages of the disease trajectory. Our aim is to review systematically the evidence concerning non-pharmacological strategies to minimise behavioural and psychological symptoms in community-dwelling older people with dementia.

**Methods/Design:**

Our approach is a two-stage co-design: a systematic mapping of the broad evidence around behavioural and psychological symptoms followed by an in-depth systematic review of studies of non-pharmacological interventions for behavioural and psychological symptoms from the perspective of their impact on community-dwelling older people with dementia and their carers. The review will include published literature involving a wide range of electronic databases using sensitive and comprehensive searches and lateral searching including checking citations.

We will produce a descriptive map of the studies by design and by the focus of interventions and apply further inclusion criteria, developed in conjunction with lay experts, to select studies for an in-depth systematic review that will include independent quality assessment and detailed data extraction by two reviewers.

The review process will be integrated with stakeholder meetings and a multidisciplinary expert advisory group to guide the review parameters and shape the research questions on the management of behavioural and psychological symptoms in people with dementia. Because studies are likely to be diverse in methodology and interventions, we will conduct a narrative synthesis of the in-depth systematic review. If appropriate, we will pool studies in a meta-analysis. We will explore review findings at both stages through focus groups and interviews with service providers, practitioners, people with dementia and carers.

**Discussion:**

This integrated review in collaboration with key stakeholders will synthesise research evidence to identify appropriate interventions for effective management of behavioural and psychological symptoms that supports people with dementia living at home and their carers, and which reflects their priorities. It will make recommendations for research and practice.

**Study registration:**

PROSPERO registration number: CRD42013004344

## Background

Two-thirds of people with dementia live at home, and for those in long-term care, they have been supported at home prior to admission. People with dementia rely on family members (carers), often with the help of social care workers and primary care services [1]. There is limited evidence to guide professionals in how to effectively support people with dementia at home and many only become aware of their circumstances following a crisis [2]. Evidence is limited on what strategies work in the home setting and what methods of on-going support are most likely to ameliorate carer strain and pre-empt unwanted residential care and/or hospitalisation [3]. In England, the National Dementia Strategy [4] and the Ministerial Advisory Group on Dementia Research [5] have called for more community-based personal support, less use of antipsychotic medication and effective management of behavioural and psychological symptoms in dementia (BPSD) to improve the quality of life for people with dementia [4,5].

### Why behavioural and psychological symptoms in dementia are important

The term BPSD is used in some healthcare settings to describe a range of symptoms that may be distressing and hard to manage for carers and professionals alike (in other settings it may be termed behaviour that challenges or distressing behaviour). These non-cognitive symptoms of BPSD are estimated to affect up to 90% of people with dementia at some stage [6,7]. Symptoms include depression, psychosis, aggression, wandering or walking/getting lost, agitation, apathy and emotional distress. Together or separately they affect the ability to sustain everyday activities, and reduce quality of life for both people with dementia and their carers [8]. The consequences of BPSD may be disturbed sleep, fatigue, increased risk of falls and accidental injuries, inadequate nutrition [9-11] and increased carer stress. Less is known about how BPSD affects people living in their own home compared to those living in long-term care settings (for example, care homes). Those experiencing BPSD are likely to have a worse prognosis, a more rapid rate of illness progression, to use more care services, and eventually move to care homes [8,12,13]. The National Institute for Clinical Excellence and Social Care Institute for Excellence dementia guidelines argue that improving cognitive or non-cognitive symptoms and maintaining day-to-day function are key to improving overall quality of life [14]. They recommend non-pharmacological management for BPSD [14,15] and highlight the limited evidence base underpinning management of BPSD (searched 2006). There is some evidence to suggest that some multi-component interventions, including psychological, behavioural and educational interventions for carers, may be effective in improving symptoms and carer well-being, but many studies lack data on BPSD, are not focused on care at home, and the evidence on cost-effectiveness is unclear [3,14,16-25]. Some systematic reviews have evaluated cognitive and non-cognitive interventions, including behaviour management therapies, that may be effective for the management of BPSD. However, although studies have been conducted in various settings, most were undertaken in long-term care facilities, such as care homes, and their possible application to home-based support remains uncertain [19,20]. These studies have reported various outcomes, and demonstrated significant clinical and methodological heterogeneity, but many are methodologically flawed. While some psychological treatments may be beneficial, their impact is not known among people living in the community [24,26-28]. Few studies consider the impact of interventions on improving daily function, or the acceptability of behaviours such as ‘safe wandering’ [6]. One recent rapid systematic overview on BPSD management [29] was limited only to reviews, included many settings and did not go back to primary studies (personal communication from author). Our study aims to extract data from all primary studies of interventions aimed at managing BPSD among people with dementia living at home as well as studies that have sought the perspectives of people with dementia and their carers.

This study will develop an up-to-date evidence base to help professionals manage BPSD symptoms among people with dementia living at home and in the community. It will take a wide definition of BPSD and further explore the impact of BPSD on activities of daily living among people with dementia, including possible difficulties with eating and drinking, continence and sleep. Our approach will identify gaps in practitioners’ understanding of the needs and wishes of people with dementia living at home, and of their carers. We will examine the evidence base with assistance from a range of stakeholders, including people with dementia and carers and service providers. Despite approaches that advocate person-centred care and the known distress posed by BPSD, it is unclear what is the best way to address and relieve these symptoms when experienced by people living in their own homes. Our study aims to highlight how responses to BPSD should be planned and offered to people with dementia living at home and their carers. We are working with an expert advisory group to guide the review parameters and lines of enquiry in the context of current evidence on BPSD.

Our research objectives are as follows:

1. To systematically map and identify the range of evidence about the management of BPSD in community-dwelling older people with dementia.

2. To conduct an in-depth systematic review to identify effective approaches to BPSD management that can promote independence and continuation of life at home.

3. To evaluate the evidence of benefit from the perspective of both people with dementia and carers.

4. To describe the suitability of the methods used to manage BPSD for people with dementia and their carers.

5. To assess the resource implications of implementing BPSD management interventions, and seek evidence of their cost-effectiveness.

6. To interpret the review’s findings with key stakeholders, including people with dementia and carers; to develop guidance and information for commissioners or service funders, care providers, practitioners, people with dementia and carers.

7. To identify gaps in evidence and recommend areas for further research.

## Methods/Design

### The review process

Our approach, guided by interviews and focus groups, is a two-stage co-design. The study involves four components:

a) A preliminary stakeholder meeting to set review parameters for the mapping.

b) Stage 1 of the integrated review is a systematic mapping of the broad literature on BPSD.

c) Stage 2 will be an in-depth systematic review of relevant sections of the evidence, appraising the outcomes, evidence of effectiveness and resource implications of non-pharmacological interventions.

d) Focus groups and interviews will be conducted after the initial mapping phase and at the end to discuss the review findings with stakeholders, in order to develop key recommendations and guidance for practice and research.

Stage 1 will address our first question: *What is the evidence for non-pharmacological interventions in the management of BPSD to support older people with dementia and their carers to live at home?* We will systematically map the available evidence by undertaking a comprehensive and systematic search of literature to identify studies on BPSD management that meet our inclusion criteria (objective 1). It will capture the breadth and heterogeneity of research on BPSD for community-dwelling older people with dementia and their carers. Studies will be categorized according to research questions, study type, methods, participants, interventions and outcomes. Stage 2 will use the findings from the mapping exercise to decide on the focus, parameters and lines of enquiry for an in-depth systematic review to meet objectives 2 to 7.

The process will be guided by stakeholder groups and a pre-established expert advisory group, which includes researchers in dementia and psychology, members of community mental health teams (dementia care nurses, consultants and clinicians), members from the Alzheimer’s Society, carer organisations, primary care teams, carer development project, social care mental health commissioning team, and carers from the Public Involvement in Research Group (based at the Centre for Research in Primary and Community Care, University of Hertfordshire). This approach has the potential to maximise lay and professional expertise and involvement in our research.

Following the identification of key questions that are focused on people with dementia living at home and their carers, we will conduct the review and synthesise the findings in collaboration with stakeholders. This process will include quality assessment, data extraction and quantitative and narrative or qualitative approaches to synthesise the findings. Both stages will include stakeholder meetings, focus groups and interviews to ensure findings reflect the priorities of people with dementia living at home and their carers.

Core to this proposal is the recognition of the need for a review method that can establish which interventions work, for whom, in what context and at what cost. It uses methods for synthesising different types of evidence that are relevant to practitioners and commissioners, and inform policy and practice [30-32]. The overall approach builds on the team’s review work on diverse sources of evidence involving stakeholders in the refinement and interpretation of the review process and in research design [14,33-38]. In addition, focus groups and interviews have been used successfully in other studies to enable stakeholders to engage in informed debate [6]. The review process will follow the established guidelines for conducting and reporting of systematic reviews [39-41].

### Stakeholder events

The review will be supported by stakeholder meetings, focus groups and interviews with carers, people with dementia, and service providers at both stages of the review process. This will directly inform the review methodology and findings through consultation with experts, including people with dementia and carers. They will be identified through the expert advisory group (members are drawn from health and social care sectors), the memory clinic (Hertfordshire Partnership NHS Trust), the Alzheimer’s Society and the patient and public involvement group of the Dementias and Neurodegenerative Disease Research Network (DeNDRoN).

#### Preliminary stakeholder meeting

Stakeholders will be asked to comment on the search strategies for identifying evidence, the appropriateness of the different kinds of review methods available to understand the evidence as well as shared terminology for challenging behaviour in dementia.

#### Stakeholder focus groups and interviews

Two focus group meetings, supplemented by interviews where participants wish, will be organised after the first stage of mapping and towards the end of the second stage of the project. Based on the findings of the preliminary mapping of the literature, the groups will be asked to identify or comment on the research on BPSD that seems important and relevant for older people with dementia living at home and their carers. The groups will also be asked to highlight the priorities and experiences of primary and social care support systems and their acceptability and accessibility. Recommendations from these meetings will help refine the focus of the in-depth review and synthesis. Once the review is complete, the groups will be invited to discuss the findings and their implications for services, education and skills development, and the on-going support of people with dementia and their carers. At the end of the consultation an accompanying consensus statement will be developed to comment on the review findings.

Participants will meet as two groups. One will be focus groups for professionals that will include health and social care practitioners, and care providers and support workers from voluntary organisations. The other will be focus groups or individual interviews for carers and people with dementia.

Topic areas for discussion will be informed by the systematic review. Participants will help identify key areas of interest and concerns around interventions for BPSD management for people with dementia living in the community. This will help further refine our research questions and inform the criteria for the in-depth systematic review.

Discussions will be taped and transcribed in full with consent of those participating. Transcripts will be independently coded by two reviewers. Discrepancies in interpretation will be discussed and the coding frame refined.

Stakeholders will ensure that the scope, key research areas and dissemination of the findings are directly informed by the priorities and interests of practitioners, people with dementia and carers, and their representatives. This approach was used by the National Institute for Clinical Excellence and Social Care Institute for Excellence guidelines [14] and methods developed by other studies and complex reviews [36-38,42]. Ethical permissions have been received for this part of the research (National Research Ethics Service Committee East of England, Cambridge Central, reference: 13/EE/0040). Written informed consent for participation in the study will be obtained from all participants.

### Review methods

#### Inclusion criteria

##### Stage 1: descriptive mapping

We will include studies that evaluate non-pharmacological strategies, programmes or interventions for managing BPSD in older people (aged 65 years and over) with dementia living at home and their carers. This will include people in sheltered housing and assisted living (in the UK referred to as sheltered housing or extra care housing) who rely on primary and social care services but will exclude those in long-term care settings, such as care homes. For the initial mapping of evidence, we will include all study types: primary studies, process and outcome evaluations, qualitative studies that are linked to outcome evaluations or are specifically related to BPSD and daily activities in people with dementia, and policy or guidance documents. All systematic reviews and relevant reviews will be screened for studies covering people with dementia living at home or in the community.

##### Stage 2: in-depth systematic review

From the broad mapping of evidence, narrower inclusion criteria will be applied to select studies for the in-depth review. At this stage we will engage with service providers, carers and people with dementia as well as the expert advisory group to determine priorities for stage 2. We expect a large map of evidence from which we are likely to include studies that specifically focus on BPSD-related interventions and support, views and experiences of carers and people with dementia related to types of support at home, and prospective designs that employ a comparison and control group with pre- and post-intervention data. Process evaluations will be examined for relevance to the interventions. We will seek to reach a consensus with the advisory group on areas most relevant for practice and development of BPSD-related interventions.

#### Identification of studies

The subject of this review covers a wide range of potentially relevant interventions. Our initial search strategies are broad but sensitive enough to ensure that we capture representative sets of a wide range of relevant studies. We have included sensitive search strategies for the large bibliographic databases and lateral searching techniques that have been shown to be important for identifying outcome evaluations [43]. The general search strategies were developed by an experienced Information Scientist with input from the research management group. These general searches will be supplemented by searches for specific interventions as required. The search terms will be adapted for use with other bibliographic databases in combination with database-specific filters where these are available (see Additional file 1 for search terms). There will be no language restrictions. Studies from main databases are being searched from January 2000 onwards. (This review will explore more recent evidence around BPSD specifically targeting the home or community setting so that findings can inform current practice. All related papers of relevant post 2000 studies will also be examined). The searches will be re-run just before the analyses and further studies retrieved for inclusion.

Searches are being conducted as follows and from clinical, psychological and sociological databases:

● Medline (PubMed), CINAHL, British Nursing Index, EMBASE, PsycInfo, DH Data, King’s Fund, Web of Science (including Science Citation Index, Social Sciences Citation Index, Arts & Humanities Citation Index, Turning Research Into Practice, and the Cochrane Library (including Cochrane Central Register of Controlled Trials (CENTRAL), Cochrane Database of Systematic Reviews, Database of Abstracts of Reviews of Effects, Health Technology Assessment, Economic, Systematic reviews, Trials, Method, Techno), The Allied and Complementary Medicine Database, Scopus

● Campbell Collaboration, ADEAR (Alzheimer’s disease clinical trials database)

● National Health Services Evidence

● Google, Google Scholar

● The Evidence for Policy and Practice Information and Co-ordinating Centre (EPPI-Centre) databases: Trials Register of Promoting Health Interventions; Database of Promoting Health Effectiveness Reviews

● Checking of reference lists from primary studies and systematic reviews (snowballing)

● Citation searches using the ‘Cited by’ option on Web of Science, Google Scholar and Scopus, and the ‘Related articles’ option on PubMed and Web of Science (‘Lateral Searching’)

● Contact with experts to uncover grey literature (for example, DeNDRoN, National Library for Health Later Life Specialist Library)

● On-going or recently completed trials and studies identified using: National Research Register (Department of Health), Social Care Research Register (Social Care Institute for Excellence); International Register of Controlled Trials, National Institutes of Health Portfolio

● The National Library of Health, Social Care Online, ALOIS (community project run by the Cochrane Dementia and Cognitive Improvement)

● NHS Economic Evaluation Database, Health Economic Evaluations Database

#### Screening

Electronic search results will be downloaded into EndNote bibliographic software. Titles and abstracts of potentially relevant citations will be screened for the broad mapping with at least 10% done independently by two reviewers against the predefined inclusion criteria. Full manuscripts of potentially relevant citations for the evidence map will be screened independently by two reviewers. At least 10% double screening will be considered if the number of papers is large. Systematic reviews and policy or practice guidance documents will be screened for primary studies. Discrepancies will be identified and resolved through discussion (with a third author where necessary).

#### Data extraction and quality assessment

For the broad mapping of evidence, data will be extracted in an Excel database to summarise the research aims, types of studies and designs, participants, interventions or approaches, settings, country, outcomes including process outcomes, barriers, and facilitators. A second reviewer will check 10% of the extracted data. Study types will be broadly classified as outcome evaluation, process evaluation, economic evaluation, methodological, intervention development, protocols, descriptive, qualitative or views study, systematic or literature reviews, and policy or guidance.

Final inclusion of studies and additional data extraction for the in-depth systematic review will be informed by the findings from the mapping and consultation with experts and stakeholders.

Detailed data for the in-depth systematic review will be extracted onto a pre-designed piloted form and will address research objectives 2 to 7. It will include study research questions, hypotheses, theoretical frameworks, sampling, settings, participants’ characteristics, intervention details, methods employed for data collection (measurement tools), methods of analysis, results including effect size estimates and times of measurement for all relevant outcomes and by subgroups (for example, gender, cultural groups), recruitment and study completion rates, acceptability to users and carers, views, barriers and facilitators from qualitative studies, providers of care, information for quality and risk of bias assessment, and authors’ conclusions. Two reviewers will extract data independently, discrepancies will be identified and resolved through discussion (with a third author where necessary). Missing data will be requested from study authors.

The quality of included studies will be assessed independently by two reviewers, using a checklist of questions. Disagreements between the reviewers on the risk of bias (quality assessment) will be resolved by discussion, with involvement of a third review author where necessary. The criteria used will be based on those of the Cochrane Collaboration and the Centre for Reviews and Dissemination guidelines for quantitative studies [40,44], and refined through piloting and agreement with the advisory group. Aspects of quality assessed will include appropriateness of study design, allocation methods, selective reporting, ascertainment of outcomes, attrition, key confounding factors, rigour of analysis, sample size and response rates.

We will use the established guidance and recommendations for missing data in a systematic review. We will contact the authors to request missing data and indicate the assumptions of any methods used to handle missing data [40,44]. We will assess the amount of missing data during data extraction and whether appropriate methods were used to account for missing data.

Our search strategy is broad, comprehensive and sensitive enough to capture representative sets of a wide range of relevant studies. To minimise publication bias, we are searching for unpublished and grey literature through contacts with experts. Our advisory group comprises members working in this field and we will explore all avenues to identify unpublished work. If appropriate, we will consult a statistician for advice on assessment of publication bias.

We will use a checklist for data extraction from qualitative studies that we know from previous experience works well [45,46]. We will examine sampling method used, types of perspectives, reporting methods and data, and whether findings are supported by data. For process evaluations we will examine the types of processes used (for example, accessibility, acceptability, appropriateness), and the intervention or delivery context. Discrepancies from independent screening, data extraction and quality assessment will be resolved through discussion and if appropriate by a third reviewer.

### Outcomes of interest

We will include the following outcomes for the descriptive map. Relevant outcomes will be informed by the evidence map and selection criteria for the in-depth systematic review.

Patient (persons with dementia) outcomes:

● Changes (reductions) in the incidence, frequency or severity of behaviour and psychological problems using standardized rating scales for BPSD or reports from carers;

● Change in level of independence (sustaining independence in activities of daily living);

● Changes in quality of life associated with BPSD and/or related problems;

● Experiences of people with dementia in relation to the effectiveness and acceptability of BPSD management and quality of life.

Carer outcomes:

● Carer strain or burden associated with managing BPSD and/or related problems;

● Carer quality of life associated with managing BPSD and/or related problems;

● Experiences of carers in relation to the effectiveness and acceptability of BPSD management and quality of life.

Organisational outcomes:

● Moves to long-term institutions or care homes;

● Delay in moving to care homes;

● Service use and costs.

Process outcomes:

● Process measures relating to BPSD management and provision of care;

● Acceptability, feasibility and satisfaction relation to BPSD management.

### Data presentation, analysis and synthesis

The final scope of the in-depth systematic review synthesis will be informed by the descriptive map and advisory group. We propose a narrative synthesis because studies are likely to be diverse in methodology and interventions. At stage 2 of the project we will be able to identify whether or not meta-analysis is appropriate, depending on the types of BPSD outcomes reported and the level of heterogeneity. The project advisory/steering group will further advise and inform this process of applying additional selection criteria for the in-depth systematic review, the types of BPSD outcomes and methods of synthesis. We will consult a statistician about the appropriateness of a meta-analysis.

To capture the diverse methodology and interventions used, results will be presented in a narrative and a tabular format. We will present a summary of the descriptive map to demonstrate the type and range of evidence according to key characteristics of the studies. For the in-depth systematic review we will present a detailed tabular summary of the characteristics of included studies, quality assessment, findings and their applicability. We will develop an analytical framework to draw out the key messages about BPSD management from the perspectives of people with dementia and their carers, to meet objectives 2 to 7.

A narrative synthesis will include ‘evidence statements’ that will summarise the results of the studies, taking into account the key issues relevant to the review questions. For an in-depth analysis of outcome evaluations, we will assess effect size estimates (overall, and according to sociodemographic variables, such as gender, ethnic or cultural subgroups, and so on) and statistical significance of the effectiveness of the intervention on each relevant outcome. Selection of outcomes will be determined by the stakeholder and advisory groups.

We will examine assessments of measures of BPSD across different countries and cultures to understand different terminologies and language around BPSD.

For qualitative studies, we will draw out key themes and concepts to identify factors associated with BPSD management, based on the most appropriate methods for the synthesis [47-49]. We will consider the findings in terms of how they might contribute to answering questions about the appropriateness of interventions, their characteristics and how they can be tailored for BPSD management, as well as provide insights into the perceptions of people with dementia and carers about BPSD and quality of life.

We will seek explanations through thematic analysis, such as how interventions might address the views and perceptions of people with dementia receiving support.

The process of thematic synthesis will be based on established methods that involve the development of descriptive themes and the generation of analytic themes [49]. These themes will then be used to identify, for example, barriers and facilitators to BPSD management and service provision. We will use software (for example, NVivo) for qualitative data analysis. The use of software may facilitate the development of themes and allow reviewers to examine the contribution made to their findings by individual studies, groups of studies, or sub-populations within studies.

#### Economic evaluation

The economic evaluation will provide information for decision makers on the value-for-money of alternative interventions [40]. Details of resources used in the delivery of interventions and costs (where reported) will be extracted from the studies included in the systematic review. In addition, evidence on the effect of interventions on use of other services will be recorded, and offsets will be explored. Findings on the resource implications of interventions will be assessed in relation to the outcomes they generate. Descriptive summaries comparing types of interventions for measures of benefit and costs will be presented, and the reliability and generalisability of findings will be discussed. Depending on the amount and range of data that are available, costs will be converted to a common date and currency for comparison. Based on previous work in the area, it is expected that only a small minority of the identified studies will be full economic evaluations (cost-effectiveness, cost utility, or cost-benefit analyses). Where these are available, data extraction will cover key methodological features, including perspective, time horizon and discounting, sources of information, measurement and valuation of resources and outcomes, and analysis of uncertainty. Quality will be assessed using a validated checklist [50].

## Discussion

This evidence synthesis integrated with findings from stakeholder groups will enable us to establish how BPSD are experienced by people with dementia and their carers living at home. It will draw out ‘what works for whom in which context’, and consider how the expertise of the carer, and the support received, influences how BPSD are recognised and managed. The review will provide a detailed account of the characteristics of components of interventions that may be effective for BPSD management and its impact on sustaining activities of daily living and, if available, factors that can inform intervention development for the effective management of BPSD. It will highlight implications for practice and evidence gaps in the research.

Outputs from the study (reports, journal papers, briefing papers, guidelines and evidence summaries) will be developed in collaboration with stakeholders to support decision making by practitioners and commissioners about services for people with dementia and their carers living at home. The findings of this study will also focus on community-based community-specific strategies and inform the development of a programme of research that can build on what is effective care and support for people with dementia who have BPSD and their carers.

## Competing interests

The authors declare that they have no competing interests.

## Authors’ contributions

DT is the principal investigator and the project lead and has written the manuscript. All authors form the Research Management Group and have contributed to the project protocol. JMcLaughlin has contributed to the searches and together with DT will conduct interviews and focus groups and data extraction. DT proposed the design of the review process and registered the protocol with PROSPERO. CG, AD, JManthorpe, SI are co-investigators and have contributed to the research questions and project design, particularly with qualitative aspects and stakeholder events. HG (co-investigator) has written the economic component of the review. AK (co-investigator) has provided clinical input and help with recruitment from the memory clinic. All authors have commented on the protocol and read and approved the final manuscript.

## Supplementary Material

Additional file 1 Search terms used in the electronic databases. Click here for file
