# Supplementary material for: A protocol for a systematic review of research on managing behavioural and psychological symptoms in dementia for community-dwelling older people: evidence mapping and syntheses
Source: Syst Rev. 2013 Aug 28;2:70. doi: 10.1186/2046-4053-2-70 (PMC3765841; doi:10.1186/2046-4053-2-70)
Supplement: Additional file 1 — Search terms used in the electronic databases. [file 2046-4053-2-70-S1.pdf]

A protocol for a systematic review of research on managing Behavioural and Psychological Symptoms in Dementia (BPSD) for community dwelling older people: evidence mapping and syntheses

Trivedi et al, 2013

Search terms used in the electronic databases

Each term is combined with ‘OR’; each section is combined with ‘AND’

Dementia, alzheimers, *AND*

non-pharmacological (non-pharmacolog*, nonpharmacolog*), non-drug, nondrug *AND*

multicomponent, multifaceted, multi-factorial, intervention, management (manage*), rehabilitation, complex, alternative, behaviour (behav*), psychological (psych*), symptom *AND*

caregivers, carers, community, community Health Services, community health planning, Community Health Services, Community Health Nursing, Community Health Workers, community living, community nursing, community dwelling, community-dwelling, day care, extra care housing, Home Care Services, home health care, home nursing, housing for the elderly, Intermediate Care Facilities, non-institutionalised, Residence Characteristics, sheltered housing ,Social Support, dwelling *AND*

activities of daily living, acupuncture, animal-assisted, aromatherapy, art therapy, Behaviour Therapy, biofeedback, Breathing Exercises, calligraphy, cognitive-behaviour, cognitive therapy, cognitive stimulation, Combined Modality Therapy, complementary therapies, counselling, creative therapy, dance, dance therapy, day care, Diet, Sodium-Restricted, reducing, Diet therapy, domestic, Electric Countershock, Electric Stimulation Therapy, electroconvulsive, exercise, exercise therapy, family therapy, group therapy, hypnosis, integrative, interview therapy, Life style, massage, music therapy, occupational therapy, Patient Education, Phototherapy, Physical Fitness, physical therapy modalities, physiotherapy, psychotherapy, complementary, reality orientation, recreation therapy, rehabilitation, relaxation, relaxation therapy, reminiscence, respite care, Sensory art therapies, Self Care, sleep therapy, support, Transcutaneous Electric Nerve Stimulation, walking, occupational therapy

Search formulations include text and subject headings for several databases.
